# Beyond nociception: rethinking pain in ovarian endometriosis a pilot study

**DOI:** 10.3389/fpain.2026.1669515

**Published:** 2026-03-11

**Authors:** Amira Quevedo, Mackenzie S. Dyrda, Terrie Vasilopoulos, Chidinma Oli, Nash S. Moawad, Roger B. Fillingim

**Affiliations:** 1 Division of Minimally Invasive Gynecologic Surgery, Department of Obstetrics and Gynecology, University of Florida College of Medicine, Gainesville, FL, United States; 2 University of Florida College of Medicine, Gainesville, FL, United States; 3 Department of Anesthesiology, University of Florida College of Medicine, Gainesville, FL, United States; 4 Department of Orthopaedic Surgery & Sports Medicine, University of Florida College of Medicine, Gainesville, FL, United States; 5 Department of Epidemiology, University of Florida College of Public Health, Gainesville, FL, United States; 6 Department of Community Dentistry and Behavioral Science, University of Florida College of Dentistry, Gainesville, FL, United States

**Keywords:** central sensitization, chronic pain, chronic pelvic pain (CPP), endometriosis, nociceptive pain, nociplastic pain

## Abstract

**Background:**

Endometriosis affects 1 in 10 women, is the most common cause of chronic pelvic pain, classically known for its nociceptive pain mechanisms. Medical therapies have limited efficacy, prevent pregnancy, and can be poorly tolerated in over 30% of cases. Meanwhile, surgical management can be associated with up to 37% of pain persistence, and over 45% undergo repeat surgery within five years.

**Objective:**

To characterize differing pain profiles between women with ovarian endometriosis and those with other benign ovarian cysts.

**Study design:**

Prospective observational cross-sectional clinical study between surgical ovarian endometriosis and control individuals. The primary outcome was to detect differences in the presence of nociplastic pain among the two groups using the Fibromyalgia (FM) Survey Score.

**Results:**

33 participants were approached from July 2024 to October 2024. Twelve participants with ovarian endometriosis and eight control participants with non-endometriosis ovarian cysts were enrolled. There was a statistically significant difference in the prevalence of elevated FM Survey score between the endometriosis group and controls (41.7% vs. 0%, *p* = 0.045). Further significant differences were identified in total FM Survey scores, Brief Pain Inventory pain severity, visual analog pain scores for dysmenorrhea, chronic pelvic pain, dyschezia, dysuria, dyspareunia, and evidence of adenomyosis.

**Conclusion:**

Ovarian endometriosis, despite its well-known inflammatory pain characteristics, is associated with elevated FM survey scores, which may suggest nociplastic centrally mediated pain mechanisms.

## Introduction

Endometriosis affects over 190 million women worldwide and is characterized by the presence of endometrial-like tissue outside the uterus and associated chronic inflammation (1, 2). It is responsible for up to 87% of chronic pelvic pain in addition to infertility, bowel obstruction, and kidney failure with detrimental impacts on quality of life (2, 3). While laparoscopic surgery alleviates pain (4), up to 37% report persistent symptoms (5), and over 45% undergo repeat surgery within five years (6). Endometriosis remains underfunded and understudied compared to male-dominant conditions (7), particularly regarding novel pain treatments (8, 9). Ovarian endometriosis, represents approximately 44% of endometriosis cases, is traditionally viewed as an inflammatory-driven subtype (10, 11), and treatment approaches target primarily nociceptive pain pathways. Yet, symptom severity does not align with disease stage (12) and 35% of asymptomatic individuals have evidence of endometriosis (13), suggesting complex pain mechanisms beyond the lesions (14, 15). Beyond nociceptive pain, some evidence suggests that nociplastic pain contributes to endometriosis-associated pain, potentially as a result of altered central pain processing (16). The International Association for the Study of Pain (IASP) has defined nociplastic pain as “pain that arises from altered nociception despite no clear evidence of actual or threatened tissue damage causing the activation of peripheral nociceptors or evidence for disease or lesion of the somatosensory system causing the pain”. These findings highlight the need for well-characterized patient pain profiles and individualized therapeutic strategies in clinical practice.

### Rationale for the current study

We hypothesize that ovarian endometriosis, known for its robust inflammatory pain profile, exhibits distinct centrally mediated pain mechanisms that can be indirectly quantified through validated pain questionnaires, compared to benign ovarian cysts in individuals without endometriosis. We aim to highlight the urgent need for the standard implementation of stratification tools for endometriosis pain phenotypes in clinical practice and ultimately inform the development of more targeted, effective treatment approaches.

## Materials and methods

This is a prospective observational cross-sectional clinical study performed in the Department of Obstetrics and Gynecology at the University of Florida Shands Hospital in Gainesville, Florida. All participants underwent written informed consent and reporting followed Strengthening the Reporting of Observational Studies in Epidemiology (STROBE) guidelines, IRB (#202300049).

### Study population

All premenopausal individuals aged 18–55 years old scheduled for laparoscopic or robotic surgery at Shand's Hospital were considered for eligibility from July 2024 to October 2024. Patients who underwent ovarian surgery were included in the study and ovarian tissue sent for pathology to confirm endometriosis based on the presence of endometrial glands and stroma. Exclusion criteria included suspected malignancy, absence of an ovarian cyst, pregnancy, breastfeeding, and immune suppression [pharmacologic or physiologic (actively taking oral steroids, transplant rejection medications, or chemotherapeutic agents)].

### Recruitment, randomization, and blinding

**Figure 1:**
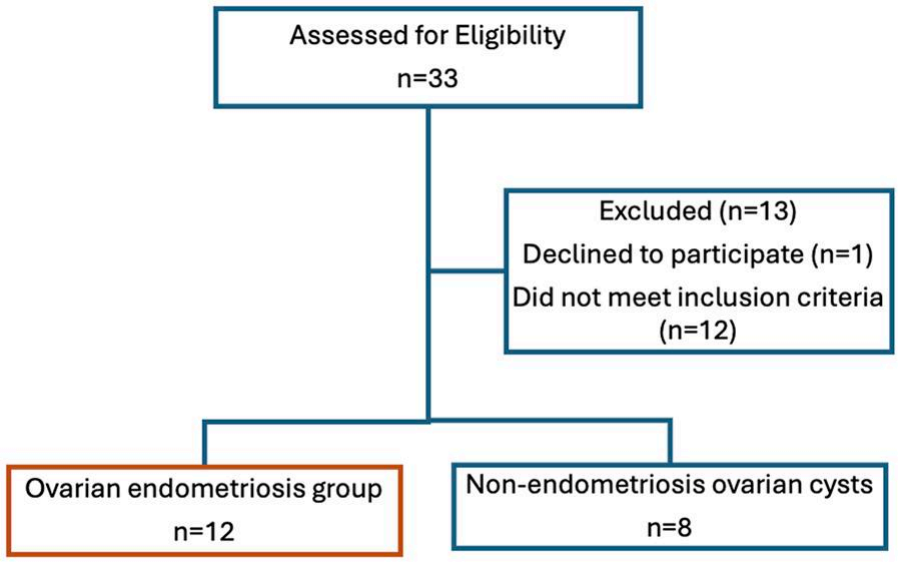
STROBE flow diagram. *STROBE*, strengthening the reporting of observational studies in epidemiology.

Eligible participants at the time of the outpatient consultation or on the day of surgery in the preoperative area at the University of Florida Health Shands Hospital were approached for informed consent. Thirty-three candidates were assessed for eligibility, 13 of whom were excluded (Figure 1), with the most common reason for their exclusion being the absence of ovarian cysts in the control group. Twenty participants met all inclusion criteria and were enrolled. The final cohort included 8 participants in the control group and 12 in the endometriosis group.

### Interventions

Participants underwent scheduled minimally invasive ovarian surgery which included ovarian cystectomy or oophorectomy depending on surgical indication. Data collected included medical and surgical history, procedure details and complications, menstrual phase at the time of surgery, medications (hormonal and analgesic), presence of chronic overlapping pain conditions (COCPs), and endometriosis stage classified using the revised American Society for Reproductive Medicine classification (rASRM) criteria. Endometriosis was confirmed by pathology. A diagnostic laparoscopy was used to rule out endometriosis in control participants.

### Endpoints

All patients received a REDCap baseline questionnaire via a QR code or web link by email. Data collected included patient reported presence of a COPC, National Institutes of Health (NIH) Helping to End Addiction Long-term® (HEAL) Initiative Common Data Elements (17) and validated endometriosis questionnaires (18) that assessed pain and its effect on quality of life included the Fibromyalgia (FM) Survey score, Brief Pain Inventory (BPI) questionnaire, a pain body map, endometriosis-associated pain on a visual analog scale (VAS) as measured in millimeters (0–100 mm), infertility diagnosis, medical and medication history. Our decision to use the FM Survey score is supported by prior studies validating its role in measuring symptoms suggestive of nociplastic pain in endometriosis and gynecologic surgery patients (19, 20). It has been postulated to measure the degree of nociplastic pain and assesses widespread pain, fatigue, sleep disturbances, and cognitive difficulties (19). It is scored from 0 to 31 with a score of 13 or greater suggesting a diagnosis of fibromyalgia and nociplastic pain mechanisms (19, 21). The BPI questionnaire was used to measure pain intensity (severity) and the impact of pain on functioning (interference) (22). The BPI instrument asks subjects to indicate the number (0, “*No Pain*” to 10, “*Pain As Bad As You Can Imagine*”) that best describes their pain on average, worst, and least in the last 24 h (22). Moreover, the BPI measures how much pain has interfered with seven daily activities, including general activity, walking, work, mood, enjoyment of life, relations with others, and sleep*,* scored as the mean of the seven interference items.

The primary outcome of the study was to detect differences in the FM Survey score at baseline between the two groups. Secondary outcomes were BPI, VAS endometriosis-associated pain scores, the presence of COPCs, and infertility.

### Statistical analysis

Measures were summarized as medians (25th to 75th percentiles) for continuous measures and as counts (percentages) for categorical measures. Group differences in continuous outcomes were evaluated using the Mann–Whitney test (non-parametric) and groups differences categorical outcomes were evaluated with Fisher's exact tests. For the primary outcome of FM Survey score, the Hodges–Lehmann (HL) estimator with 95% confidence intervals (95%CIs) was additionally calculated to assess median of groups differences. Because data did not meet normality assumptions of parametric tests, non-parametric tests were used. Analyses were performed using statistical package JMP Pro 18 (SAS Institute Inc, Cary, NC).

## Results

For this pilot study a sample size of 20 (12 cases vs. 8 controls) will provide reliable estimates of means and variances necessary to 1) establish the premise that the FM Survey Score will be different among those with endometriosis, and controls 2) to appropriately perform a power analysis for a larger study to confirm the preliminary results we expect to observe in this pilot study (23). The study began enrollment on July 26, 2024 (Figure 1).

Baseline characteristics of the study groups are reported in Table 1. Patients with endometriosis were slightly older and had a higher representation of Latino patients. The groups were similar in terms of race and current medication usage. The most common birth control reported was progestin-only pills (4/20, 25%), followed by progestin intrauterine devices (2/20,10%). A quarter (25%) of the sample reported uses of acetaminophen (*n* = 5) and non-steroidal anti-inflammatory medications (*n* = 5), respectively. Among both cohorts, menstrual phase distribution and the proportion of participants using hormonal suppression did not differ significantly (Table 2). Within the group with endometriosis 25% were in the menstrual phase, 25% were receiving hormonally suppression, 8.3% were in the ovulatory phase, and 41.7% were in proliferative phase. Among participants in the control group, 37.5% were hormonally suppressed, 12.5% were in premenstrual phase, 37.5% were in ovulatory phase, and 12.5% were in proliferative phase. Overall, Fischer's exact test indicated no significant group differences (*p* = 0.143).

**Table 1:** Baseline sample characteristics.

Demographics	Total sample *n* = 20	Endometriosis *n* = 12	Control *n* = 8
Age, median years (25th, 75th percentile)	38.5 (33.5, 45.5)	40.5 (33.5, 43.5)	35.5 (32.75, 47)
White/Caucasian, *n* (%)	12 (60.0%)	8 (66.7%)	4 (50.0%)
Latino, *n* (%)		4 (36.4%)	0 (0%)
Currently taking pain medications, birth control, or a hormonal medication, *n* (%)	13 (65.0%)	8 (66.7%)	5 (62.5%)

**Table 2:** Group comparison of the menstrual phase showing no significant differences. Fischer's exact test was *p* = 0.143.

Phase	Endo *n* = 12	Control *n* = 8	Z-score	*p*-value
Menstrual	25%	0%	1.53	0.126
Hormonally suppressed	25%	37.5%	0.6	0.550
Pre-menstrual	0%	12.5%	1.26	0.209
Ovulation	8.3%	37.5%	1.6	0.110
Proliferative	41.7%	12.5%	1.4	0.163

### Primary endpoint

When comparing control and endometriosis participants, statistically significant differences in FM Survey score of 6.5 (3–8) vs. 11.5 (10–19.75) *p* = (0.0025) were identified, respectively. The median of the differences was significantly greater than 0; HL = 8.0 (95% CIs: 3.0, 13.0). The proportion of individuals with an elevated FM Survey Score in the control group was 0% vs. 41.7% in the endometriosis group (*p* = 0.045).

### Secondary endpoints

**Figure 2:**
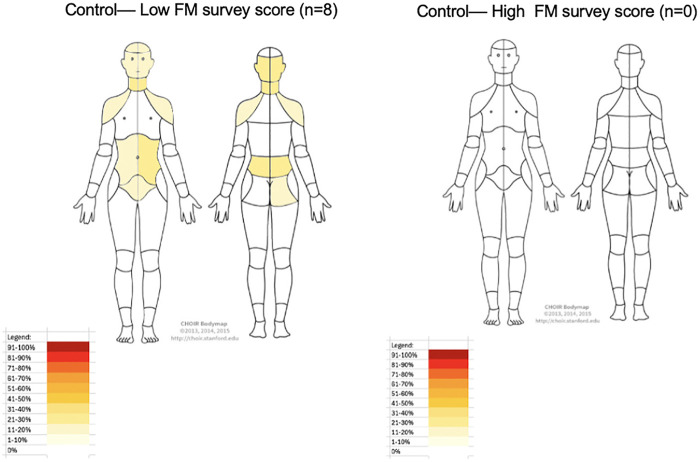
Heat pain body map showing pain distribution for control participants with low FM survey scores and absence of high FM survey scores control participants. *FM survey score*, Fibromyalgia survey score.

**Figure 3:**
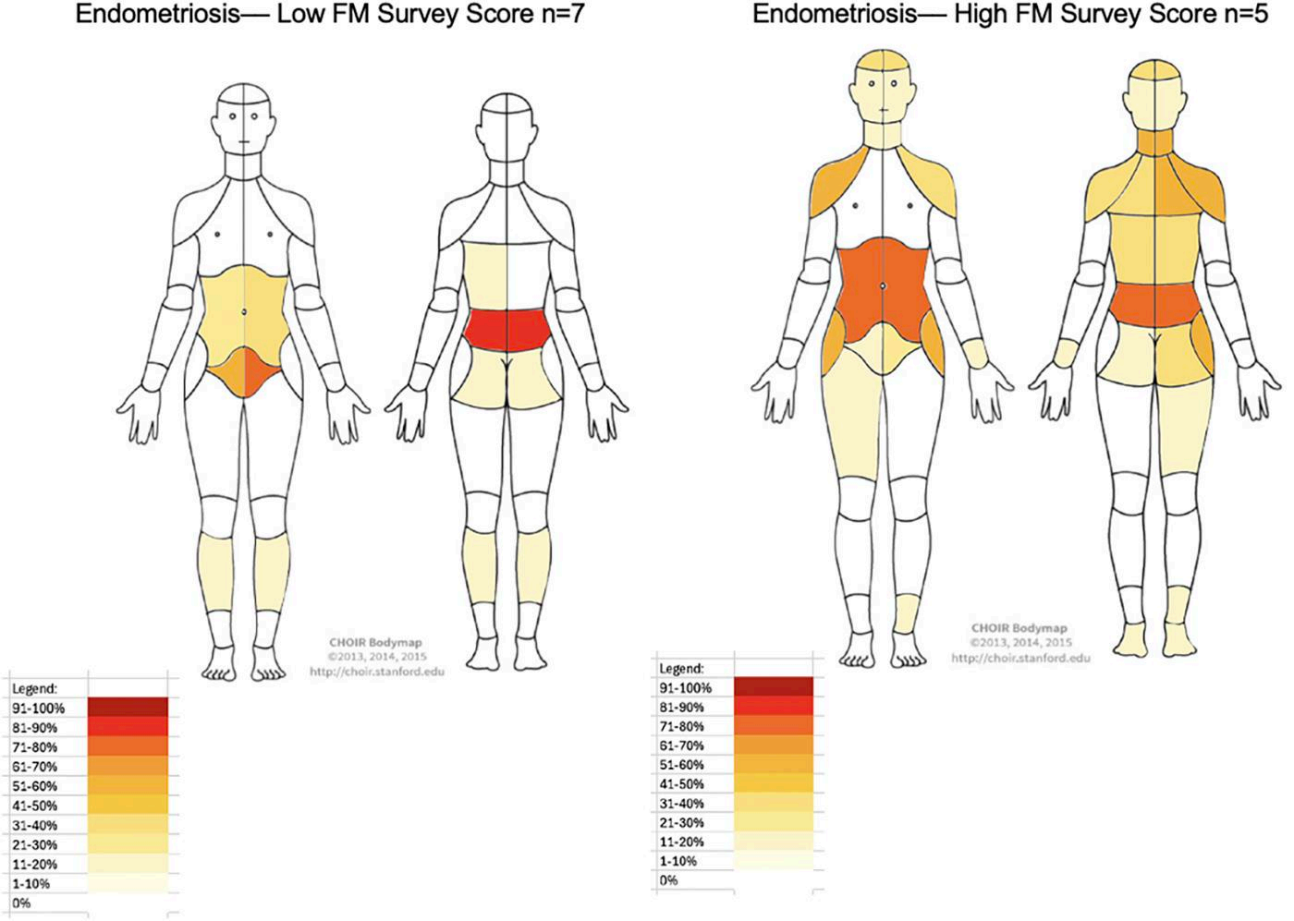
Heat pain body map of endometriosis participants with low and high FM survey scores. *FM survey score*, Fibromyalgia survey score.

When comparing secondary outcomes control vs. endometriosis showed statistically significant differences in VAS scores: chronic pelvic pain 0.5 (0–2) vs. 4 (2.25–5) (*p* = 0.041), dysmenorrhea 3 (3–3.75) vs. 9 (8.25–10) (*p* < 0.001), dyschezia 0 (0–1.75) vs. 7 (5–9) (*p* < 0.001), dysuria 0 vs. 3.5 (0–6.5) (*p* = 0.011), and deep dyspareunia 0.5 (0–5.75) vs. 7 (3.25–9) (*p* = 0.019). Adenomyosis was co-prevalent in 12.5% of controls and 75% of ovarian endometriosis cases (*p* = 0.020). Adenomyosis was diagnosed by the Morphological Uterus Sonographic Assessment (MUSA) imaging criteria (24) or uterine pathology at the time of hysterectomy. Deep infiltrating endometriosis was found in 83.3% of participants with endometriosis. BPI was voluntarily filled out by 15/20 patients with significant differences between control and endometriosis cases in BPI pain severity 2.38 (0.25–3.75) vs. 6 (3.25–7.25), respectively, *p* = 0.026 (Table 3). There were no significant differences between controls and endometriosis cases with respect to BPI pain interference, infertility, chronic overlapping pain conditions, uterine myomas, or anxiety and depression. Figures 2, 3 highlights the pain distribution reported by all participants according to their FM Survey score.

**Table 3:** Secondary outcomes.

Secondary outcomes	Endometriosis *n* = 12	Control *n* = 8	*p*-value
Brief Pain Inventory Pain Severity, median (25th, 75th percentile)	6.0 (3.25, 7.25)	2.38 (0.25, 3.75)	0.026
Visual Analog Scales, median (25th, 75th percentile)			
Chronic Pelvic Pain	4 (2.25, 5.0)	0.5 (0, 5.75)	0.041
Dysmenorrhea	9 (8.25, 10.0)	3 (3, 3.75)	<0.001
Dyschezia	7 (5.0, 9.0)	0 (0,1.0)	<0.001
Dysuria	3.5 (0, 6.5)	0 (0,0)	0.011
Deep Dyspareunia	7.0 (3.25, 9.0)	0.5 (0.0, 5.75)	
Adenomyosis, *n* (%)	9 (75.0%)	1 (12.5%)	0.020
Chronic Overlapping Pain Conditions, *n* (%)			
Migraine or Tension Headaches	4 (33.3%)	2 (25.0%)	1.0
Interstitial Cystitis	2 (16.7%)	0 (0%)	0.495
Irritable Bowel Syndrome	1 (8.3%)	0 (0%)	1.0
Pelvic Inflammatory Disease	0 (0%)	0 (0%)	-
Myofascial Pain Dysfunction	1 (8.3%)	1 (12.5%)	1.0
Fibromyalgia	0 (0%)	0 (0%)	-
Uterine Fibroids, *n* (%)	7 (58.3%)	4 (50.0%)	1.0
Anxiety or Panic Attacks, *n* (%)	4 (33.3%)	2 (25.0%)	1.0
Depression, *n* (%)	4 (33.3%)	2 (25.0%)	1.0
Infertility, *n* (%)			
Difficulty Getting Pregnant in Last Year	3 (25.0%)	2 (25.0%)	1.0
Received Infertility Treatment (IVF, Clomid, Letrozole, IUI, Hormone Injections)	1 (8.3%)	2 (25.0%)	0.537

### Safety

Overall, surgery was well tolerated. In the endometriosis group, one participant was admitted postoperatively for suspected pelvic infection on POD #8 and was managed with intravenous antibiotics. No major Clavien-Dindo grade 3–5 complications were seen in either group.

## Discussion

In this prospective, observational clinical study, participants undergoing ovarian endometriosis surgery showed a statistically significant difference in FM Survey scores when compared to control ovarian cysts. This is the first study to report findings that may support centrally mediated pain mechanisms specifically in ovarian endometriosis. Further significant differences were seen in our secondary outcomes, namely, BPI pain severity, VAS pain scores, and co-existence of adenomyosis.

### Clinical implications

Endometriosis affects approximately 10% of reproductive-aged women worldwide. Treatments are limited in endometriosis and focus on nociceptive pain modulation. This study's findings challenge the conventional view of ovarian endometriosis pain as primarily a nociceptive condition, and reveals findings which may indicate undiagnosed nociplastic pain, in over 40% of participants based on FM survey scores. This mirrors persistent pain rates exceeding 37% after endometriosis surgery (5). This highlights the need for further research into centrally acting mechanisms of pain in ovarian endometriosis. This is thematically in agreement with prior studies that have observed common nerve pathways innervating colon, bladder, and reproductive organs which contribute to cross-organ pain sensitization in endometriosis (34). When looking at all endometriosis types (superficial, deep, and ovarian) with chronic pelvic pain prior studies observed decreased gray matter volume in key neural pain processing regions such as the thalamus, cingulate gyrus, putamen, and insula (16). Overall, other authors have proposed increasing clinical trials investigating the effectiveness of existing and novel centrally acting medications (25–27). Currently, these treatments are often used only after multiple failed medical and surgical interventions. Tools like the Chronic Overlapping Pain Condition-Screener, can support earlier identification of patients most at risk of underdiagnosis, leading to personalized care (28, 29). We further observed increased widespread pain, as assessed by a self-reported pain body map, among participants with endometriosis (Figure 3) and elevated FM survey scores compared to controls. Participants with widespread pain consistently reported painful bladder and bowel symptoms, suggesting sensitization within shared peripheral neural pathways, including contributions from the pelvic hypogastric nerves. Collectively, these findings support the presence of a prevalent, centrally mediated pain phenotype that extends beyond pain localized to inflammatory endometriosis lesions. The high prevalence and cost associated with treatment, and high surgical recidivism for pain in ovarian endometriosis signify an unmet need that mandates further research into the pathogenesis and pain biologic mechanisms. Implementing validated assessments like the FM Survey and a pain body map in clinical practice has the potential to enhance the standard of care in endometriosis.

### Strengths and limitations

Our publication has several strengths including collection of validated pain questionnaires, confirmatory surgical diagnosis of endometriosis types, and collection of variables such as menstrual phase, hormonal medications, and presence of COPCs. Conversely, we cannot determine the contributions of existing deep infiltrating endometriosis and COPCs on FM survey scores. This high prevalence of deep endometriosis is in agreement with other publications reporting an 87.6% of coexisting deep endometriosis with ovarian endometriosis (30). Likewise, menstrual cycle and hormonal influences on pain sensitivity have been reported, yet the magnitude of these associations is highly variable (31). Moreover, numerous publications (32, 33) have found differences in pain responses depending on menstrual cycle, with worsening pain reported during the menstrual and premenstrual phase. Although, there were no statistically significant differences between the two cohorts with regards to menstrual phase, 25% of participants with endometriosis were in the menstrual phase at the time of the questionnaire completion, raising the possibility that phase related fluctuations in pain physiology may skew reported pain levels. Participant pain questionnaires, including the BPI and FM Survey are limited by recall bias and lack mechanistic specificity. Furthermore, not all participants completed the voluntary pain questionnaires. Although, an elevated FM survey score may suggest nociplastic pain mechanisms we cannot definitively prove these mechanisms are of central origin. Additionally, this pilot study was specific to ovarian endometriosis with a low sample size, limiting statistical power.

## Conclusion

We report a significant difference in FM survey scores possibly suggesting the presence of nociplastic pain in participants with ovarian endometriosis compared to control ovarian cysts. Larger studies are necessary to confirm our findings along with randomized clinical trials to determine the efficacy of centrally acting medications in addition to standard surgical and medical treatments in endometriosis to effect pain.

## Data Availability

The raw data supporting the conclusions of this article will be made available by the authors, without undue reservation.

## References

[1] Koninckx PR Ussia A Tahlak M Adamyan L Wattiez A Martin DC Infection as a potential cofactor in the genetic-epigenetic pathophysiology of endometriosis: a systematic review. Facts Views Vis Obgyn. (2019) 11(3):209–16. PMCID: PMC7020943. 32082526 PMC7020943

[2] Zondervan KT Becker CM Missmer SA. Endometriosis. N Engl J Med. (2020) 382(13):1244–56. 10.1056/NEJMra1810764 32212520

[3] Ling FW. Randomized controlled trial of depot leuprolide in patients with chronic pelvic pain and clinically suspected endometriosis. Pelvic Pain Study Group. Obstet Gynecol. (1999) 93(1):51–8. 10.1016/s0029-7844(98)00341-x 9916956

[4] Quevedo A Parikh S Reinstine J Chamseddine P Gaskins JT Whalen C The impact of metronidazole on pain persistence after fertility-sparing endometriosis surgery: METROFERT randomized study. Am J Obstet Gynecol. (2025) 232(1):106.e1–e9. 10.1016/j.ajog.2024.07.006 39019388

[5] Sutton CJ Ewen SP Whitelaw N Haines P. Prospective, randomized, double-blind, controlled trial of laser laparoscopy in the treatment of pelvic pain associated with minimal, mild, and moderate endometriosis. Fertil Steril. (1994) 62(4):696–700. 10.1016/s0015-0282(16)56990-8 7926075

[6] Shakiba K Bena JF McGill KM Minger J Falcone T. Surgical treatment of endometriosis: a 7-year follow-up on the requirement for further surgery. Obstet Gynecol. (2008) 111(6):1285–92. 10.1097/AOG.0b013e3181758ec6. Erratum in: *Obstet Gynecol*. (2008) **112**(3):710. 18515510

[7] Mirin AA. Gender disparity in the funding of diseases by the U.S. national institutes of health. J Womens Health. (2021) 30(7):956–63. 10.1089/jwh.2020.8682 PMC8290307 33232627

[8] As-Sanie S Black R Giudice LC Gray Valbrun T Gupta J Jones B Assessing research gaps and unmet needs in endometriosis. Am J Obstet Gynecol. (2019) 221(2):86–94. 10.1016/j.ajog.2019.02.033 30790565

[9] Buggio L Dridi D Barbara G Merli CEM Cetera GE Vercellini P. Novel pharmacological therapies for the treatment of endometriosis. Expert Rev Clin Pharmacol. (2022) 15(9):1039–52. 10.1080/17512433.2022.2117155 36000243

[10] Busacca M Vignali M. Endometrioma excision and ovarian reserve: a dangerous relation. J Minim Invasive Gynecol. (2009) 16(2):142–8. 10.1016/j.jmig.2008.12.013 19249702

[11] Zhang J Zhao W Zhou Y Xi S Xu X Du X Pyroptotic T cell-derived active IL-16 has a driving function in ovarian endometriosis development. Cell Rep Med. (2024) 5(3):101476. 10.1016/j.xcrm.2024.101476 38508138 PMC10983113

[12] Koninckx PR Ussia A Mashiach R Vilos G Martin DC. Endometriosis can cause pain at a distance. J Obstet Gynaecol Can. (2021) 43(9):1035–6. 10.1016/j.jogc.2021.06.002 34481578

[13] Nnoaham KE Hummelshoj L Webster P d'Hooghe T de Cicco Nardone F de Cicco Nardone C Impact of endometriosis on quality of life and work productivity: a multicenter study across ten countries. Fertil Steril. (2011) 96(2):366–373.e8. 10.1016/j.fertnstert.2011.05.090 21718982 PMC3679489

[14] Bartley EJ Alappattu MJ Manko K Lewis H Vasilopoulos T Lamvu G. Presence of endometriosis and chronic overlapping pain conditions negatively impacts the pain experience in women with chronic pelvic–abdominal pain: a cross-sectional survey. Women’s Health. (2024) 20:17455057241248017. 10.1177/17455057241248017 PMC11057341 38682290

[15] Raimondo D Raffone A Renzulli F Sanna G Raspollini A Bertoldo L Prevalence and risk factors of central sensitization in women with endometriosis. J Minim Invasive Gynecol. (2023) 30(1):73–80.e1. 10.1016/j.jmig.2022.10.007 36441085

[16] As-Sanie S Kim J Schmidt-Wilcke T Sundgren PC Clauw DJ Napadow V Functional connectivity is associated with altered brain chemistry in women with endometriosis-associated chronic pelvic pain. J Pain. (2016) 17(1):1–13. 10.1016/j.jpain.2015.09.008 26456676 PMC4698023

[17] Adams MCB Hassett AL Clauw DJ Hurley RW. The NIH HEAL pain common data elements (CDE): a great start but a long way to the finish line. Pain Med. (2025) 26(3):146–55. 10.1093/pm/pnae110 39495148 PMC11879210

[18] Abbott J Billow M Gallant T Hackett L Kho RM Knapman B Patient-reported outcome measures used in randomized controlled trials following surgical intervention for endometriosis: a structured review from the AAGL practice guidelines group. J Minim Invasive Gynecol. (2024) 31(2):71–83.e17. 10.1016/j.jmig.2023.10.017 37931893

[19] Till SR Schrepf A Clauw DJ Harte SE Williams DA As-Sanie S. Association between nociplastic pain and pain severity and impact in women with chronic pelvic pain. J Pain. (2023) 24(8):1406–14. 10.1016/j.jpain.2023.03.004 36958459 PMC10511662

[20] As-Sanie S Till SR Schrepf AD Griffith KC Tsodikov A Missmer SA Incidence and predictors of persistent pelvic pain following hysterectomy in women with chronic pelvic pain. Am J Obstet Gynecol. (2021) 225(5):568.e1–568.e11. 10.1016/j.ajog.2021.08.038 34464585 PMC9297195

[21] Kaplan CM Kelleher E Irani A Schrepf A Clauw DJ Harte SE. Deciphering nociplastic pain: clinical features, risk factors and potential mechanisms. Nat Rev Neurol. (2024) 20(6):347–63. Nature Research. 10.1038/s41582-024-00966-8 38755449

[22] Atkinson TM Mendoza TR Sit L Passik S Scher HI Cleeland C The brief pain inventory and its “pain at its worst in the last 24 hours” item: clinical trial endpoint considerations. Pain Med. (2010) 11(3):337–46. 10.1111/j.1526-4637.2009.00774.x 20030743 PMC3806650

[23] Moore CG Carter RE Nietert PJ Stewart PW. Recommendations for planning pilot studies in clinical and translational research. Clin Transl Sci. (2011) 4(5):332–7. 10.1111/j.1752-8062.2011.00347 22029804 PMC3203750

[24] Harmsen MJ Van den Bosch T de Leeuw RA Dueholm M Exacoustos C Valentin L Consensus on revised definitions of morphological uterus sonographic assessment (MUSA) features of adenomyosis: results of modified Delphi procedure. Ultrasound Obstet Gynecol. (2022) 60(1):118–31. 10.1002/uog.24786 34587658 PMC9328356

[25] Andrade MA Soares LC De Oliveira MAP. The effect of neuromodulatory drugs on the intensity of chronic pelvic pain in women: a systematic review. Rev Bras Ginecol Obstet. (2022) 44(9):891–8. 10.1055/s-0042-1755459 36044916 PMC9948135

[26] Cheong YC Smotra G Williams ACDC. Non-surgical interventions for the management of chronic pelvic pain. Cochrane Database Syst Rev. (2014) 2014(3):CD008797. 10.1002/14651858.CD008797.pub2 24595586 PMC10981791

[27] Marchand G Masoud AT Govindan M Ware K King A Ruther S Systematic review and meta-analysis of the efficacy of gabapentin in chronic female pelvic pain without another diagnosis. AJOG Global Rep. (2022) 2(1):100042. 10.1016/j.xagr.2021.100042 PMC9563541 36274967

[28] Fillingim R Ohrbach R Greenspan J Sanders A Rathnayaka N Maixner W Associations of psychologic factors with multiple chronic overlapping pain conditions. J Oral Facial Pain Headache. (2020) 34:s85–s100. 10.11607/ofph.2584 32975543 PMC10165716

[29] Schrepf A Maixner W Fillingim R Veasley C Ohrbach R Smith S The chronic overlapping pain condition screener. J Pain. (2024) 25(1):265–72. 10.1016/j.jpain.2023.08.009 37633574 PMC13390051

[30] Bindra V Naem A Swetha P Kahla J Reddy A Laganà AS Mapping deep endometriosis in patients with ovarian endometriomas according to the #Enzian classification: a single-center retrospective analysis. Front Med. (2025) 12:1626445. 10.3389/fmed.2025.1626445 PMC12331578 40786093

[31] Fillingim RB King CD Ribeiro-Dasilva MC Rahim-Williams B Riley JL 3rd. Sex, gender, and pain: a review of recent clinical and experimental findings. J Pain. (2009) 10(5):447–85. 10.1016/j.jpain.2008.12.001 19411059 PMC2677686

[32] Fortún-Rabadán R Boudreau SA Bellosta-López P Herrero P Graven-Nielsen T Doménech-García V. Facilitated central pain mechanisms across the menstrual cycle in dysmenorrhea and enlarged pain distribution in women with Longer pain history. J Pain. (2023) 24(9):1541–54. 10.1016/j.jpain.2023.04.005 37100358

[33] Rezaii T Hirschberg AL Carlström K Ernberg M. The influence of menstrual phases on pain modulation in healthy women. J Pain. (2012) 13(7):646–55. 10.1016/j.jpain.2012.04.002 22634142

[34] Maddern J Grundy L Castro J Brierley SM. Pain in endometriosis. Front Cell Neurosci. (2020) 14:590823. 10.3389/fncel.2020.590823 33132854 PMC7573391

